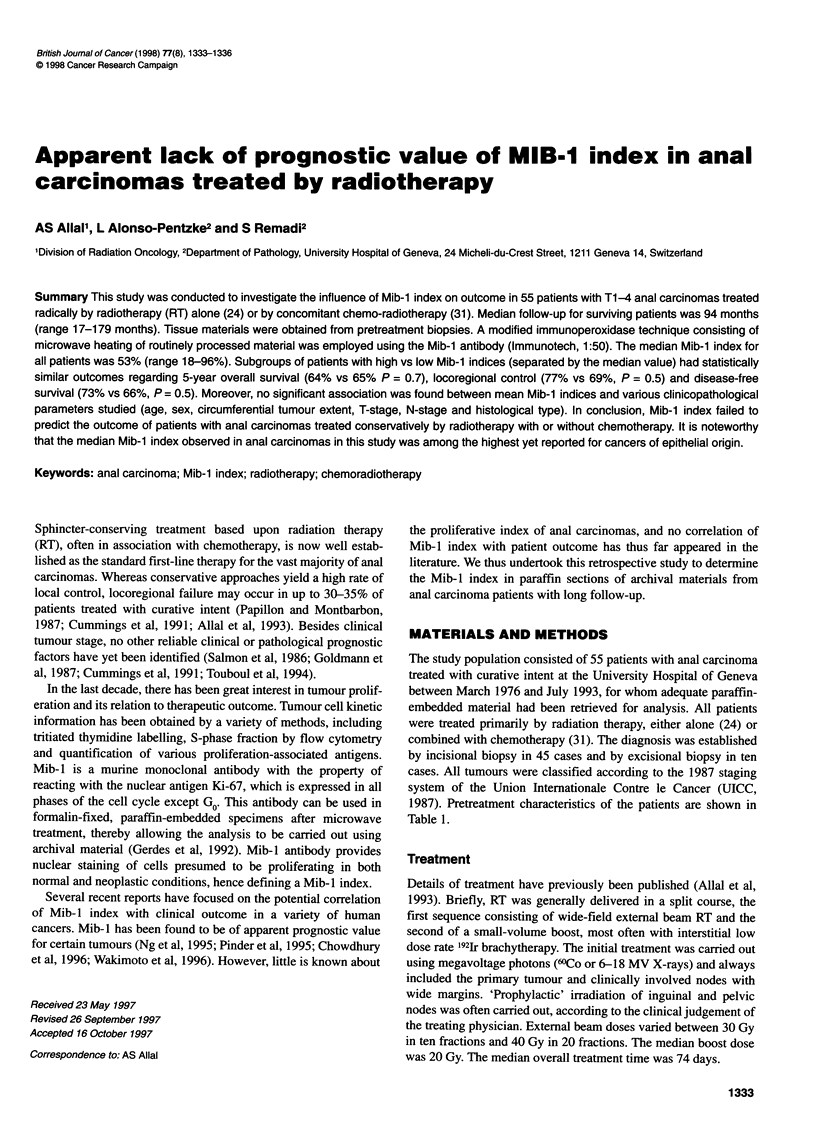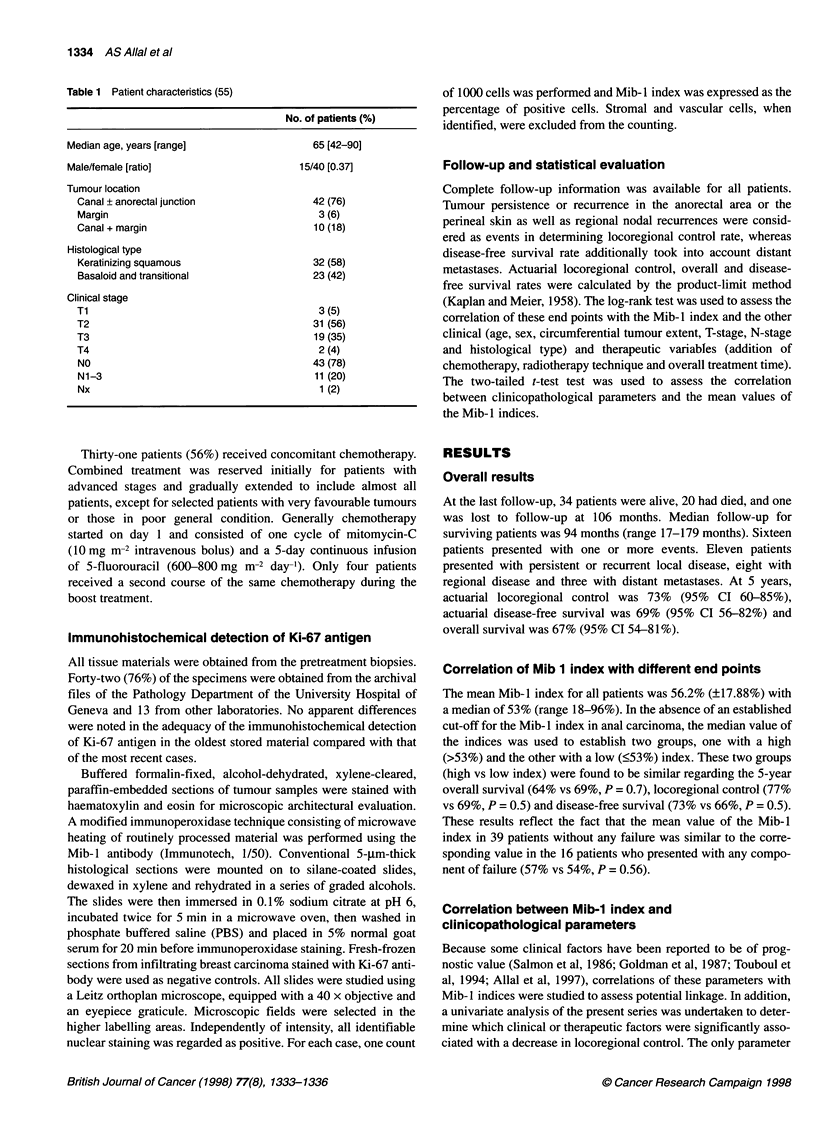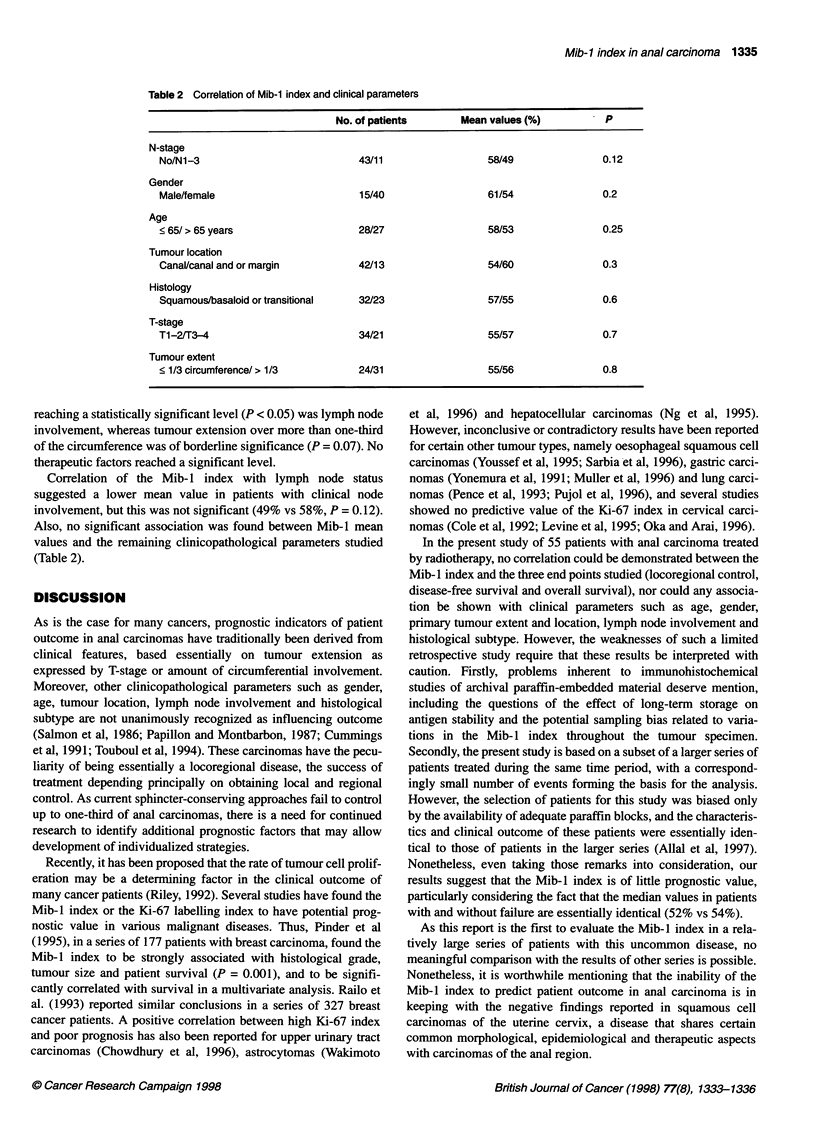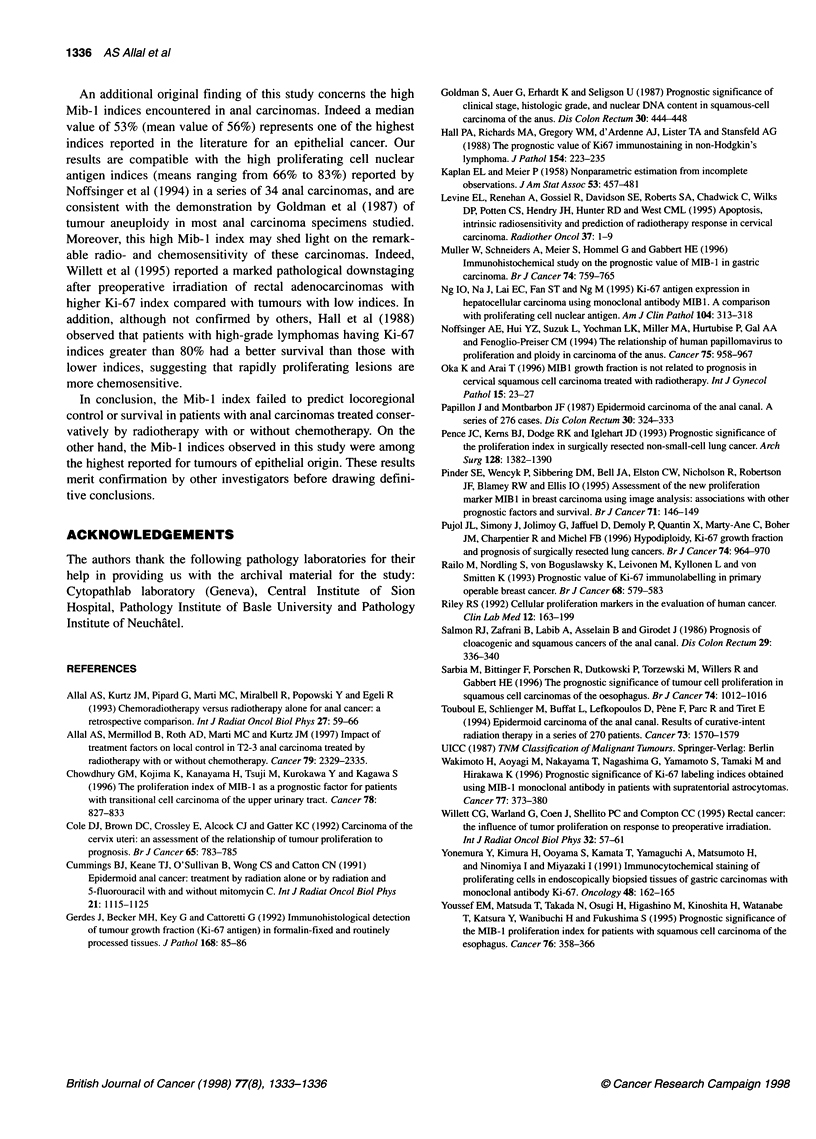# Apparent lack of prognostic value of MIB-1 index in anal carcinomas treated by radiotherapy.

**DOI:** 10.1038/bjc.1998.222

**Published:** 1998-04

**Authors:** A. S. Allal, L. Alonso-Pentzke, S. Remadi

**Affiliations:** Division of Radiation Oncology, University Hospital of Geneva, Switzerland.

## Abstract

This study was conducted to investigate the influence of Mib-1 index on outcome in 55 patients with T1-4 anal carcinomas treated radically by radiotherapy (RT) alone (24) or by concomitant chemo-radiotherapy (31). Median follow-up for surviving patients was 94 months (range 17-179 months). Tissue materials were obtained from pretreatment biopsies. A modified immunoperoxidase technique consisting of microwave heating of routinely processed material was employed using the Mib-1 antibody (Immunotech, 1:50). The median Mib-1 index for all patients was 53% (range 18-96%). Subgroups of patients with high vs low Mib-1 indices (separated by the median value) had statistically similar outcomes regarding 5-year overall survival (64% vs 65% P = 0.7), locoregional control (77% vs 69%, P = 0.5) and disease-free survival (73% vs 66%, P = 0.5). Moreover, no significant association was found between mean Mib-1 indices and various clinicopathological parameters studied (age, sex, circumferential tumour extent, T-stage, N-stage and histological type). In conclusion, Mib-1 index failed to predict the outcome of patients with anal carcinomas treated conservatively by radiotherapy with or without chemotherapy. It is noteworthy that the median Mib-1 index observed in anal carcinomas in this study was among the highest yet reported for cancers of epithelial origin.


*British Journal of Cancer* (1998) **77**(8), 1333–1336
© 1998 Cancer Research Campaign

### Apparent lack of prognostic value of MIB-1 index in anal carcinomas treated by radiotherapy

**AS Allal[1], L Alonso-Pentzke[2] and S Remadi[2]**

[1]Division of Radiation Oncology, [2]Department of Pathology, University Hospital of Geneva, 24 Micheli-du-Crest Street, 1211 Geneva 14, Switzerland

**Summary** This study was conducted to investigate the influence of Mib-1 index on outcome in 55 patients with T1–4 anal carcinomas treated radically by radiotherapy (RT) alone (24) or by concomitant chemo-radiotherapy (31). Median follow-up for surviving patients was 94 months (range 17–179 months). Tissue materials were obtained from pretreatment biopsies. A modified immunoperoxidase technique consisting of microwave heating of routinely processed material was employed using the Mib-1 antibody (Immunotech, 1:50). The median Mib-1 index for all patients was 53% (range 18–96%). Subgroups of patients with high vs low Mib-1 indices (separated by the median value) had statistically similar outcomes regarding 5-year overall survival (64% vs 65% $P$ = 0.7), locoregional control (77% vs 69%, $P$ = 0.5) and disease-free survival (73% vs 66%, $P$ = 0.5). Moreover, no significant association was found between mean Mib-1 indices and various clinicopathological parameters studied (age, sex, circumferential tumour extent, T-stage, N-stage and histological type). In conclusion, Mib-1 index failed to predict the outcome of patients with anal carcinomas treated conservatively by radiotherapy with or without chemotherapy. It is noteworthy that the median Mib-1 index observed in anal carcinomas in this study was among the highest yet reported for cancers of epithelial origin.

**Keywords:** anal carcinoma; Mib-1 index; radiotherapy; chemoradiotherapy

Sphincter-conserving treatment based upon radiation therapy (RT), often in association with chemotherapy, is now well established as the standard first-line therapy for the vast majority of anal carcinomas. Whereas conservative approaches yield a high rate of local control, locoregional failure may occur in up to 30–35% of patients treated with curative intent (Papillon and Montbarbon, 1987; Cummings et al, 1991; Allal et al, 1993). Besides clinical tumour stage, no other reliable clinical or pathological prognostic factors have yet been identified (Salmon et al, 1986; Goldmann et al, 1987; Cummings et al, 1991; Touboul et al, 1994).

In the last decade, there has been great interest in tumour proliferation and its relation to therapeutic outcome. Tumour cell kinetic information has been obtained by a variety of methods, including tritiated thymidine labelling, S-phase fraction by flow cytometry and quantification of various proliferation-associated antigens. Mib-1 is a murine monoclonal antibody with the property of reacting with the nuclear antigen Ki-67, which is expressed in all phases of the cell cycle except $G_0$. This antibody can be used in formalin-fixed, paraffin-embedded specimens after microwave treatment, thereby allowing the analysis to be carried out using archival material (Gerdes et al, 1992). Mib-1 antibody provides nuclear staining of cells presumed to be proliferating in both normal and neoplastic conditions, hence defining a Mib-1 index.

Several recent reports have focused on the potential correlation of Mib-1 index with clinical outcome in a variety of human cancers. Mib-1 has been found to be of apparent prognostic value for certain tumours (Ng et al, 1995; Pinder et al, 1995; Chowdhury et al, 1996; Wakimoto et al, 1996). However, little is known about the proliferative index of anal carcinomas, and no correlation of Mib-1 index with patient outcome has thus far appeared in the literature. We thus undertook this retrospective study to determine the Mib-1 index in paraffin sections of archival materials from anal carcinoma patients with long follow-up.

*Received 23 May 1997*
*Revised 26 September 1997*
*Accepted 16 October 1997*

*Correspondence to:* AS Allal

### MATERIALS AND METHODS

The study population consisted of 55 patients with anal carcinoma treated with curative intent at the University Hospital of Geneva between March 1976 and July 1993, for whom adequate paraffin-embedded material had been retrieved for analysis. All patients were treated primarily by radiation therapy, either alone (24) or combined with chemotherapy (31). The diagnosis was established by incisional biopsy in 45 cases and by excisional biopsy in ten cases. All tumours were classified according to the 1987 staging system of the Union Internationale Contre le Cancer (UICC, 1987). Pretreatment characteristics of the patients are shown in Table 1.

### Treatment

Details of treatment have previously been published (Allal et al, 1993). Briefly, RT was generally delivered in a split course, the first sequence consisting of wide-field external beam RT and the second of a small-volume boost, most often with interstitial low dose rate $^{192}$Ir brachytherapy. The initial treatment was carried out using megavoltage photons ($^{60}$Co or 6–18 MV X-rays) and always included the primary tumour and clinically involved nodes with wide margins. 'Prophylactic' irradiation of inguinal and pelvic nodes was often carried out, according to the clinical judgement of the treating physician. External beam doses varied between 30 Gy in ten fractions and 40 Gy in 20 fractions. The median boost dose was 20 Gy. The median overall treatment time was 74 days.

**Table 1** Patient characteristics (55)

| | No. of patients (%) |
|---|---|
| Median age, years [range] | 65 [42–90] |
| Male/female [ratio] | 15/40 [0.37] |
| Tumour location | |
| Canal ± anorectal junction | 42 (76) |
| Margin | 3 (6) |
| Canal + margin | 10 (18) |
| Histological type | |
| Keratinizing squamous | 32 (58) |
| Basaloid and transitional | 23 (42) |
| Clinical stage | |
| T1 | 3 (5) |
| T2 | 31 (56) |
| T3 | 19 (35) |
| T4 | 2 (4) |
| N0 | 43 (78) |
| N1–3 | 11 (20) |
| Nx | 1 (2) |

Thirty-one patients (56%) received concomitant chemotherapy. Combined treatment was reserved initially for patients with advanced stages and gradually extended to include almost all patients, except for selected patients with very favourable tumours or those in poor general condition. Generally chemotherapy started on day 1 and consisted of one cycle of mitomycin-C (10 mg $m^{-2}$ intravenous bolus) and a 5-day continuous infusion of 5-fluorouracil (600–800 mg $m^{-2}$ $day^{-1}$). Only four patients received a second course of the same chemotherapy during the boost treatment.

### Immunohistochemical detection of Ki-67 antigen

All tissue materials were obtained from the pretreatment biopsies. Forty-two (76%) of the specimens were obtained from the archival files of the Pathology Department of the University Hospital of Geneva and 13 from other laboratories. No apparent differences were noted in the adequacy of the immunohistochemical detection of Ki-67 antigen in the oldest stored material compared with that of the most recent cases.

Buffered formalin-fixed, alcohol-dehydrated, xylene-cleared, paraffin-embedded sections of tumour samples were stained with haematoxylin and eosin for microscopic architectural evaluation. A modified immunoperoxidase technique consisting of microwave heating of routinely processed material was performed using the Mib-1 antibody (Immunotech, 1/50). Conventional 5-µm-thick histological sections were mounted on to silane-coated slides, dewaxed in xylene and rehydrated in a series of graded alcohols. The slides were then immersed in 0.1% sodium citrate at pH 6, incubated twice for 5 min in a microwave oven, then washed in phosphate buffered saline (PBS) and placed in 5% normal goat serum for 20 min before immunoperoxidase staining. Fresh-frozen sections from infiltrating breast carcinoma stained with Ki-67 antibody were used as negative controls. All slides were studied using a Leitz orthoplan microscope, equipped with a 40 × objective and an eyepiece graticule. Microscopic fields were selected in the higher labelling areas. Independently of intensity, all identifiable nuclear staining was regarded as positive. For each case, one count of 1000 cells was performed and Mib-1 index was expressed as the percentage of positive cells. Stromal and vascular cells, when identified, were excluded from the counting.

### Follow-up and statistical evaluation

Complete follow-up information was available for all patients. Tumour persistence or recurrence in the anorectal area or the perineal skin as well as regional nodal recurrences were considered as events in determining locoregional control rate, whereas disease-free survival rate additionally took into account distant metastases. Actuarial locoregional control, overall and disease-free survival rates were calculated by the product-limit method (Kaplan and Meier, 1958). The log-rank test was used to assess the correlation of these end points with the Mib-1 index and the other clinical (age, sex, circumferential tumour extent, T-stage, N-stage and histological type) and therapeutic variables (addition of chemotherapy, radiotherapy technique and overall treatment time). The two-tailed *t*-test test was used to assess the correlation between clinicopathological parameters and the mean values of the Mib-1 indices.

### RESULTS

### Overall results

At the last follow-up, 34 patients were alive, 20 had died, and one was lost to follow-up at 106 months. Median follow-up for surviving patients was 94 months (range 17–179 months). Sixteen patients presented with one or more events. Eleven patients presented with persistent or recurrent local disease, eight with regional disease and three with distant metastases. At 5 years, actuarial locoregional control was 73% (95% CI 60–85%), actuarial disease-free survival was 69% (95% CI 56–82%) and overall survival was 67% (95% CI 54–81%).

### Correlation of Mib 1 index with different end points

The mean Mib-1 index for all patients was 56.2% (±17.88%) with a median of 53% (range 18–96%). In the absence of an established cut-off for the Mib-1 index in anal carcinoma, the median value of the indices was used to establish two groups, one with a high (>53%) and the other with a low (≤53%) index. These two groups (high vs low index) were found to be similar regarding the 5-year overall survival (64% vs 69%, $P = 0.7$), locoregional control (77% vs 69%, $P = 0.5$) and disease-free survival (73% vs 66%, $P = 0.5$). These results reflect the fact that the mean value of the Mib-1 index in 39 patients without any failure was similar to the corresponding value in the 16 patients who presented with any component of failure (57% vs 54%, $P = 0.56$).

### Correlation between Mib-1 index and clinicopathological parameters

Because some clinical factors have been reported to be of prognostic value (Salmon et al, 1986; Goldman et al, 1987; Touboul et al, 1994; Allal et al, 1997), correlations of these parameters with Mib-1 indices were studied to assess potential linkage. In addition, a univariate analysis of the present series was undertaken to determine which clinical or therapeutic factors were significantly associated with a decrease in locoregional control. The only parameter

© Cancer Research Campaign 1998

**Table 2** Correlation of Mib-1 index and clinical parameters

| | No. of patients | Mean values (%) | P |
|---|---|---|---|
| N-stage | | | |
| No/N1–3 | 43/11 | 58/49 | 0.12 |
| Gender | | | |
| Male/female | 15/40 | 61/54 | 0.2 |
| Age | | | |
| ≤ 65/ > 65 years | 28/27 | 58/53 | 0.25 |
| Tumour location | | | |
| Canal/canal and or margin | 42/13 | 54/60 | 0.3 |
| Histology | | | |
| Squamous/basaloid or transitional | 32/23 | 57/55 | 0.6 |
| T-stage | | | |
| T1–2/T3–4 | 34/21 | 55/57 | 0.7 |
| Tumour extent | | | |
| ≤ 1/3 circumference/ > 1/3 | 24/31 | 55/56 | 0.8 |

reaching a statistically significant level ($P < 0.05$) was lymph node involvement, whereas tumour extension over more than one-third of the circumference was of borderline significance ($P = 0.07$). No therapeutic factors reached a significant level.

Correlation of the Mib-1 index with lymph node status suggested a lower mean value in patients with clinical node involvement, but this was not significant (49% vs 58%, $P = 0.12$). Also, no significant association was found between Mib-1 mean values and the remaining clinicopathological parameters studied (Table 2).

### DISCUSSION

As is the case for many cancers, prognostic indicators of patient outcome in anal carcinomas have traditionally been derived from clinical features, based essentially on tumour extension as expressed by T-stage or amount of circumferential involvement. Moreover, other clinicopathological parameters such as gender, age, tumour location, lymph node involvement and histological subtype are not unanimously recognized as influencing outcome (Salmon et al, 1986; Papillon and Montbarbon, 1987; Cummings et al, 1991; Touboul et al, 1994). These carcinomas have the peculiarity of being essentially a locoregional disease, the success of treatment depending principally on obtaining local and regional control. As current sphincter-conserving approaches fail to control up to one-third of anal carcinomas, there is a need for continued research to identify additional prognostic factors that may allow development of individualized strategies.

Recently, it has been proposed that the rate of tumour cell proliferation may be a determining factor in the clinical outcome of many cancer patients (Riley, 1992). Several studies have found the Mib-1 index or the Ki-67 labelling index to have potential prognostic value in various malignant diseases. Thus, Pinder et al (1995), in a series of 177 patients with breast carcinoma, found the Mib-1 index to be strongly associated with histological grade, tumour size and patient survival ($P = 0.001$), and to be significantly correlated with survival in a multivariate analysis. Railo et al. (1993) reported similar conclusions in a series of 327 breast cancer patients. A positive correlation between high Ki-67 index and poor prognosis has also been reported for upper urinary tract carcinomas (Chowdhury et al, 1996), astrocytomas (Wakimoto et al, 1996) and hepatocellular carcinomas (Ng et al, 1995). However, inconclusive or contradictory results have been reported for certain other tumour types, namely oesophageal squamous cell carcinomas (Youssef et al, 1995; Sarbia et al, 1996), gastric carcinomas (Yonemura et al, 1991; Muller et al, 1996) and lung carcinomas (Pence et al, 1993; Pujol et al, 1996), and several studies showed no predictive value of the Ki-67 index in cervical carcinomas (Cole et al, 1992; Levine et al, 1995; Oka and Arai, 1996).

In the present study of 55 patients with anal carcinoma treated by radiotherapy, no correlation could be demonstrated between the Mib-1 index and the three end points studied (locoregional control, disease-free survival and overall survival), nor could any association be shown with clinical parameters such as age, gender, primary tumour extent and location, lymph node involvement and histological subtype. However, the weaknesses of such a limited retrospective study require that these results be interpreted with caution. Firstly, problems inherent to immunohistochemical studies of archival paraffin-embedded material deserve mention, including the questions of the effect of long-term storage on antigen stability and the potential sampling bias related to variations in the Mib-1 index throughout the tumour specimen. Secondly, the present study is based on a subset of a larger series of patients treated during the same time period, with a correspondingly small number of events forming the basis for the analysis. However, the selection of patients for this study was biased only by the availability of adequate paraffin blocks, and the characteristics and clinical outcome of these patients were essentially identical to those of patients in the larger series (Allal et al, 1997). Nonetheless, even taking those remarks into consideration, our results suggest that the Mib-1 index is of little prognostic value, particularly considering the fact that the median values in patients with and without failure are essentially identical (52% vs 54%).

As this report is the first to evaluate the Mib-1 index in a relatively large series of patients with this uncommon disease, no meaningful comparison with the results of other series is possible. Nonetheless, it is worthwhile mentioning that the inability of the Mib-1 index to predict patient outcome in anal carcinoma is in keeping with the negative findings reported in squamous cell carcinomas of the uterine cervix, a disease that shares certain common morphological, epidemiological and therapeutic aspects with carcinomas of the anal region.

© Cancer Research Campaign 1998 

An additional original finding of this study concerns the high Mib-1 indices encountered in anal carcinomas. Indeed a median value of 53% (mean value of 56%) represents one of the highest indices reported in the literature for an epithelial cancer. Our results are compatible with the high proliferating cell nuclear antigen indices (means ranging from 66% to 83%) reported by Noffsinger et al (1994) in a series of 34 anal carcinomas, and are consistent with the demonstration by Goldman et al (1987) of tumour aneuploidy in most anal carcinoma specimens studied. Moreover, this high Mib-1 index may shed light on the remarkable radio- and chemosensitivity of these carcinomas. Indeed, Willett et al (1995) reported a marked pathological downstaging after preoperative irradiation of rectal adenocarcinomas with higher Ki-67 index compared with tumours with low indices. In addition, although not confirmed by others, Hall et al (1988) observed that patients with high-grade lymphomas having Ki-67 indices greater than 80% had a better survival than those with lower indices, suggesting that rapidly proliferating lesions are more chemosensitive.

In conclusion, the Mib-1 index failed to predict locoregional control or survival in patients with anal carcinomas treated conservatively by radiotherapy with or without chemotherapy. On the other hand, the Mib-1 indices observed in this study were among the highest reported for tumours of epithelial origin. These results merit confirmation by other investigators before drawing definitive conclusions.

### ACKNOWLEDGEMENTS

The authors thank the following pathology laboratories for their help in providing us with the archival material for the study: Cytopathlab laboratory (Geneva), Central Institute of Sion Hospital, Pathology Institute of Basle University and Pathology Institute of Neuchâtel.

### REFERENCES

Allal AS, Kurtz JM, Pipard G, Marti MC, Miralbell R, Popowski Y and Egeli R (1993) Chemoradiotherapy versus radiotherapy alone for anal cancer: a retrospective comparison. *Int J Radiat Oncol Biol Phys* **27**: 59–66

Allal AS, Mermillod B, Roth AD, Marti MC and Kurtz JM (1997) Impact of treatment factors on local control in T2-3 anal carcinoma treated by radiotherapy with or without chemotherapy. *Cancer* **79**: 2329–2335.

Chowdhury GM, Kojima K, Kanayama H, Tsuji M, Kurokawa Y and Kagawa S (1996) The proliferation index of MIB-1 as a prognostic factor for patients with transitional cell carcinoma of the upper urinary tract. *Cancer* **78**: 827–833

Cole DJ, Brown DC, Crossley E, Alcock CJ and Gatter KC (1992) Carcinoma of the cervix uteri: an assessment of the relationship of tumour proliferation to prognosis. *Br J Cancer* **65**: 783–785

Cummings BJ, Keane TJ, O'Sullivan B, Wong CS and Catton CN (1991) Epidermoid anal cancer: treatment by radiation alone or by radiation and 5-fluorouracil with and without mitomycin C. *Int J Radiat Oncol Biol Phys* **21**: 1115–1125

Gerdes J, Becker MH, Key G and Cattoretti G (1992) Immunohistological detection of tumour growth fraction (Ki-67 antigen) in formalin-fixed and routinely processed tissues. *J Pathol* **168**: 85–86

Goldman S, Auer G, Erhardt K and Seligson U (1987) Prognostic significance of clinical stage, histologic grade, and nuclear DNA content in squamous-cell carcinoma of the anus. *Dis Colon Rectum* **30**: 444–448

Hall PA, Richards MA, Gregory WM, d'Ardenne AJ, Lister TA and Stansfeld AG (1988) The prognostic value of Ki67 immunostaining in non-Hodgkin's lymphoma. *J Pathol* **154**: 223–235

Kaplan EL and Meier P (1958) Nonparametric estimation from incomplete observations. *J Am Stat Assoc* **53**: 457–481

Levine EL, Renehan A, Gossiel R, Davidson SE, Roberts SA, Chadwick C, Wilks DP, Potten CS, Hendry JH, Hunter RD and West CML (1995) Apoptosis, intrinsic radiosensitivity and prediction of radiotherapy response in cervical carcinoma. *Radiother Oncol* **37**: 1–9

Muller W, Schneiders A, Meier S, Hommel G and Gabbert HE (1996) Immunohistochemical study on the prognostic value of MIB-1 in gastric carcinoma. *Br J Cancer* **74**: 759–765

Ng IO, Na J, Lai EC, Fan ST and Ng M (1995) Ki-67 antigen expression in hepatocellular carcinoma using monoclonal antibody MIB1. A comparison with proliferating cell nuclear antigen. *Am J Clin Pathol* **104**: 313–318

Noffsinger AE, Hui YZ, Suzuk L, Yochman LK, Miller MA, Hurtubise P, Gal AA and Fenoglio-Preiser CM (1994) The relationship of human papillomavirus to proliferation and ploidy in carcinoma of the anus. *Cancer* **75**: 958–967

Oka K and Arai T (1996) MIB1 growth fraction is not related to prognosis in cervical squamous cell carcinoma treated with radiotherapy. *Int J Gynecol Pathol* **15**: 23–27

Papillon J and Montbarbon JF (1987) Epidermoid carcinoma of the anal canal. A series of 276 cases. *Dis Colon Rectum* **30**: 324–333

Pence JC, Kerns BJ, Dodge RK and Iglehart JD (1993) Prognostic significance of the proliferation index in surgically resected non-small-cell lung cancer. *Arch Surg* **128**: 1382–1390

Pinder SE, Wencyk P, Sibbering DM, Bell JA, Elston CW, Nicholson R, Robertson JF, Blamey RW and Ellis IO (1995) Assessment of the new proliferation marker MIB1 in breast carcinoma using image analysis: associations with other prognostic factors and survival. *Br J Cancer* **71**: 146–149

Pujol JL, Simony J, Jolimoy G, Jaffuel D, Demoly P, Quantin X, Marty-Ane C, Boher JM, Charpentier R and Michel FB (1996) Hypodiploidy, Ki-67 growth fraction and prognosis of surgically resected lung cancers. *Br J Cancer* **74**: 964–970

Railo M, Nordling S, von Boguslawsky K, Leivonen M, Kyllonen L and von Smitten K (1993) Prognostic value of Ki-67 immunolabelling in primary operable breast cancer. *Br J Cancer* **68**: 579–583

Riley RS (1992) Cellular proliferation markers in the evaluation of human cancer. *Clin Lab Med* **12**: 163–199

Salmon RJ, Zafrani B, Labib A, Asselain B and Girodet J (1986) Prognosis of cloacogenic and squamous cancers of the anal canal. *Dis Colon Rectum* **29**: 336–340

Sarbia M, Bittinger F, Porschen R, Dutkowski P, Torzewski M, Willers R and Gabbert HE (1996) The prognostic significance of tumour cell proliferation in squamous cell carcinomas of the oesophagus. *Br J Cancer* **74**: 1012–1016

Touboul E, Schlienger M, Buffat L, Lefkopoulos D, Pène F, Parc R and Tiret E (1994) Epidermoid carcinoma of the anal canal. Results of curative-intent radiation therapy in a series of 270 patients. *Cancer* **73**: 1570–1579

UICC (1987) *TNM Classification of Malignant Tumours*. Springer-Verlag: Berlin

Wakimoto H, Aoyagi M, Nakayama T, Nagashima G, Yamamoto S, Tamaki M and Hirakawa K (1996) Prognostic significance of Ki-67 labeling indices obtained using MIB-1 monoclonal antibody in patients with supratentorial astrocytomas. *Cancer* **77**: 373–380

Willett CG, Warland G, Coen J, Shellito PC and Compton CC (1995) Rectal cancer: the influence of tumor proliferation on response to preoperative irradiation. *Int J Radiat Oncol Biol Phys* **32**: 57–61

Yonemura Y, Kimura H, Ooyama S, Kamata T, Yamaguchi A, Matsumoto H, and Ninomiya I and Miyazaki I (1991) Immunocytochemical staining of proliferating cells in endoscopically biopsied tissues of gastric carcinomas with monoclonal antibody Ki-67. *Oncology* **48**: 162–165

Youssef EM, Matsuda T, Takada N, Osugi H, Higashino M, Kinoshita H, Watanabe T, Katsura Y, Wanibuchi H and Fukushima S (1995) Prognostic significance of the MIB-1 proliferation index for patients with squamous cell carcinoma of the esophagus. *Cancer* **76**: 358–366

© Cancer Research Campaign 1998